# Parametric Study of Reinforced Concrete Columns Embedded with PET Bottles Under Compression

**DOI:** 10.3390/ma19132865

**Published:** 2026-07-04

**Authors:** Sadiq Al Bayati, Sami W. Tabsh

**Affiliations:** Civil Engineering Department, American University of Sharjah, Sharjah 26666, United Arab Emirates; b00082494@alumni.aus.edu

**Keywords:** PET, voids, reinforced concrete, columns, axial load, sustainability, finite element modeling, nonlinear analysis, parametric study

## Abstract

This study presents finite element investigations on the use of polyethylene terephthalate (PET) bottles as void formers in reinforced concrete columns subjected to pure axial compression. The incorporation of PET bottles in reinforced concrete structures reduces concrete consumption while also providing a sustainable disposal solution for used PET bottles. In addition, for the same amount of concrete used in equivalent solid and voided columns, the voided column can exhibit greater flexural capacity due to the increased moment arm of the longitudinal reinforcement. In this study, a finite element-based parametric study is conducted on reinforced concrete columns containing voids within the core. The model was validated using the results of an experimental testing program consisting of 16 scaled reinforced concrete columns, of which 8 specimens had solid cross-sections and 8 specimens had hollow cross-sections. The numerical study considered variations in the longitudinal reinforcement ratio, tie spacing, concrete compressive strength and shape of cross-section. The finite element analyses were conducted using ABAQUS with consideration of material nonlinearity, and the results showed good agreement with the experimental findings. Furthermore, a parametric study was performed to quantify the effects of concrete compressive strength, longitudinal and transverse reinforcement ratios, void diameter, and column cross-sectional dimensions on the load-carrying capacity, ductility, stiffness, and residual strength of the columns. The findings of the study demonstrated that introducing voids formed by tightly stacked PET bottles within the core of reinforced concrete tied columns does not adversely affect their structural behavior under axial compressive loading, as the response of the voided columns was found to be comparable to that of their solid counterparts. The parametric study demonstrated that concrete compressive strength and longitudinal reinforcement ratio significantly influenced the load-carrying capacity and stiffness of voided columns, whereas tie spacing and void diameter had comparatively moderate effects, and changing the column shape while maintaining the same cross-sectional area as the equivalent solid section had negligible influence on the overall structural performance.

## 1. Introduction

Concrete remains the dominant construction material worldwide because of its versatility, ease of application, and the widespread availability of its raw ingredients [1]. Despite these advantages, its large-scale production has significant environmental consequences. In particular, the manufacture of cement—the primary binding component of concrete—is a major source of greenhouse gas emissions, contributing substantial amounts of carbon dioxide as well as other pollutants, including nitrous oxide and sulfur compounds [2]. These gases contribute to various forms of pollution, including visual and noise, and can cause numerous adverse health effects while significantly contributing to global warming [3]. Concrete production also contributes to water pollution and solid waste. To elaborate further, water used to clean equipment is often discharged into setting ponds, leading to contamination, and concrete itself constitutes a large portion of demolition and construction waste which often ends up in landfills [4]. The global population continues to grow at an unprecedented pace, driving an increasing demand for infrastructure development. As a result, concrete production is expected to expand accordingly, further intensifying the environmental impact associated with its manufacture. Consequently, emissions of greenhouse gases and other harmful pollutants are projected to increase unless more sustainable construction practices are adopted [5].

In addition to the environmental impacts associated with concrete production, plastic waste has become another critical global challenge. Worldwide plastic production has increased dramatically over recent decades, reaching approximately 335 million tons in 2016 [6]. The accumulation of non-biodegradable plastic products, particularly single-use bottles, presents serious environmental risks. Rather than decomposing naturally, plastics gradually fragment into microscopic particles known as microplastics, a process that may take several centuries or even millennia. These particles persist in terrestrial and aquatic ecosystems, while improperly discarded plastic waste threatens wildlife through ingestion and entanglement, especially in marine environments [7]. Furthermore, the degradation of plastic materials can release hazardous chemical compounds, including potentially carcinogenic substances, thereby contributing to long-term environmental pollution.

Columns are among the primary load-bearing elements in reinforced concrete structures, transferring loads from the floors and roof to the foundation. They are vertical structural members characterized by relatively small cross-sectional dimensions compared with their height and are designed to resist predominantly compressive forces, which may be accompanied by bending moments. Depending on architectural and structural requirements, columns may be constructed with square, rectangular, circular, or other cross-sectional configurations. To ensure adequate strength, ductility, and overall structural performance, concrete columns are reinforced with both longitudinal and transverse steel reinforcement. Longitudinal reinforcement enhances the axial and flexural load-carrying capacities, while transverse reinforcement, typically in the form of ties, confines the concrete core, improves shear resistance and ductility, and restrains the longitudinal bars against outward buckling following spalling of the concrete cover.

To reduce concrete consumption and the associated environmental burden of cement production, several researchers have explored the introduction of internal voids within reinforced concrete members. Among the various approaches, the use of discarded single-use plastic bottles as void formers remains relatively unexplored. Existing studies have predominantly concentrated on incorporating polyethylene terephthalate (PET) in the form of fibers, whereas the application of PET bottles to create internal voids has received comparatively little attention. Consequently, the present study seeks to address this research gap. A comprehensive review of the available literature, with particular emphasis on analytical and finite element investigations, identified only a small number of studies examining this innovative approach.

For example, Zayan et al. [8] applied this concept to create voids in glass fiber-reinforced polymer (GFRP)-reinforced one-way concrete slabs, thereby reducing the self-weight while having only a minimal effect on the load-carrying capacity. In a similar study on steel-reinforced concrete slabs, Sabbar et al. [9] utilized polyethylene terephthalate (PET) bottles and steel meshes to fabricate and test one-way voided concrete slabs. Their results showed that the load–displacement responses of the voided slabs were nearly identical to those of their equivalent solid slabs, with comparable ultimate load capacities.

In an earlier investigation conducted under the supervision of the second author of the present study, Hassan [10] examined the shear performance of one-way reinforced concrete slabs containing internal voids formed by PET bottles. The experimental program comprised 13 full-scale, shear-critical slab specimens, each 600 mm wide, tested under three-point bending. In addition, an analytical model was formulated to estimate the shear capacity and subsequently validated using a database of 55 slab specimens reported in the literature. The predicted voided-to-solid shear strength ratio ranged from 0.61 to 1.04, while the corresponding shear stress ratio varied between 0.82 and 1.29. The findings demonstrated that the voided slabs exhibited structural behavior comparable to that of conventional solid slabs, with no significant loss in ductility. Moreover, changes in concrete compressive strength and longitudinal reinforcement ratio had only a limited effect on the shear resistance of the voided specimens. A follow-up study by AlHomsi [11] investigated the flexural behavior of reinforced concrete one-way slabs incorporating PET bottle voids. The study examined eleven reinforced concrete slabs, each 2200 mm long and 600 mm wide, instrumented with strain gauges and LVDTs and tested under four-point bending using displacement-controlled loading. Although these studies employed the same voiding concept proposed in the present research, they focused on reinforced concrete slabs rather than columns and primarily investigated the concept through experimental testing instead of numerical analysis.

Alajrameh et al. [12] conducted a comprehensive review comparing the structural response of solid and equivalent hollow reinforced concrete columns. Their assessment showed that hollow columns generally possess lower load-carrying capacity and reduced displacement capacity after yielding of the reinforcement because of the absence of a fully confined concrete core. The lack of internal concrete also results in nonuniform confinement, as the lateral dilation induced by axial compression produces biaxial stress conditions within the remaining concrete wall. Despite these differences, the review reported that both solid and hollow confined columns reached similar axial strains at failure, whereas the lateral expansion of hollow columns was approximately one-quarter that of comparable solid columns. The influence of transverse confinement on circular hollow reinforced concrete columns was investigated numerically by Liang and Sritharan [13] using the finite element method. Their three-dimensional nonlinear model incorporated the effects of confining pressure and concrete dilation to establish appropriate stress–strain relationships for the confined concrete core. The analyses indicated that the highest confinement efficiency was achieved using two layers of transverse reinforcement connected by cross ties when the wall thickness ratio ranged from 0.125 to 0.20. In contrast, for sections with a wall thickness ratio of 0.10, a single confinement layer was found to be more practical because an additional layer provided only marginal improvements while increasing reinforcement congestion. Under this configuration, the confined concrete strength was only 3.6% lower than that of an equivalent solid column. Ma et al. [14] performed a finite element investigation of hollow reinforced concrete columns encased in steel tubes to improve the structural behavior of concrete-filled steel tube members. The numerical study examined the influence of steel yield strength, tube wall thickness, concrete strength, concrete wall thickness, and longitudinal reinforcement diameter. The results demonstrated consistent improvements in the ultimate load-carrying capacity as each of these parameters increased, confirming the effectiveness of the proposed composite section.

Yang and Okumus [15] evaluated the structural behavior of rectangular hollow columns constructed with ultra-high-performance concrete (UHPC) and high-strength steel using finite element, moment–curvature, and flexure–shear analyses. Compared with conventional concrete columns, UHPC specimens exhibited a shallower neutral axis at failure because of their higher compressive strength. Nevertheless, for the same level of confinement, failure was governed more frequently by crushing of the concrete core than by fracture of the longitudinal reinforcement. The use of high-strength steel increased both the allowable concrete strain and the flexural resistance of the columns. Based on these findings, the authors concluded that UHPC is a suitable material for hollow rectangular bridge columns where web shear is critical and that high-strength steel further enhances performance under high axial loads. The incorporation of waste plastic in cementitious materials has also been widely investigated. Al-Hadithi et al. [16] examined the influence of waste plastic fibers (WPF) on the fresh and mechanical properties of concrete by testing nine mixtures with fiber volume fractions of 0.5%, 0.75%, and 1% and aspect ratios of 15, 30, and 45. The inclusion of WPF reduced the dry density, ultrasonic pulse velocity, and thermal conductivity by approximately 9%, 14%, and 19%, respectively. In contrast, post-cracking behavior, ductility, and impact resistance improved considerably, with the optimum performance obtained for specimens containing 1% fibers with an aspect ratio of 45. Foti [17] investigated the use of recycled PET bottle fibers produced through simple mechanical cutting without additional processing. The study demonstrated that even a relatively small quantity of recycled PET fibers substantially enhanced the post-cracking behavior of plain concrete. Although both lamellar and closed-loop (“O”-shaped) fibers increased toughness, the closed-loop configuration produced superior performance because its geometry provided more effective crack-bridging action.

The feasibility of utilizing complete PET bottles as construction materials was explored experimentally by Avila et al. [18]. Their investigation considered bottles filled with either sand or plastic waste, tested both as individual building units and when embedded within concrete elements. The results confirmed that incorporating PET bottles into concrete members represents a practical and sustainable construction alternative. Similarly, Safinia and Alkalbani [19] evaluated the use of recycled PET water bottles as internal void formers in concrete masonry blocks. Their experimental results indicated that blocks containing PET bottle voids achieved compressive strengths approximately 57% higher than those of conventional locally manufactured masonry units. Waroonkun et al. [20] studied concrete blocks incorporating PET bottle flakes as partial aggregate replacement. They concluded that the optimum mixture for non-load-bearing wall applications consisted of a cement-to-aggregate ratio of 1:3, with the aggregate composed of 20% PET flakes and 80% sand and a water-to-cement ratio of 0.50. Finally, Askar et al. [21] presented a comprehensive review of PET applications in concrete. Their survey highlighted that most published studies have focused on the use of PET fibers rather than intact PET bottles. The review further reported that incorporating PET fibers can increase the splitting tensile strength of concrete by approximately 10–20%, reduce permeability by up to 5%, and improve compressive strength by about 5%.

## 2. Research Objectives and Scope

This study proposes a sustainable construction approach in which non-biodegradable polyethylene terephthalate (PET) bottles are embedded within reinforced concrete structural elements subjected to axial compression, thereby providing a permanent disposal solution for plastic waste. This novel concept offers several environmental and structural benefits, including reduced concrete consumption and lower carbon dioxide emissions associated with cement production. The proposed system is simple to implement, cost-effective, and particularly well suited for developing regions due to its sustainability and economic advantages.

The study investigates the axial compressive load–deflection behavior of reinforced concrete columns with solid cross-sections and compares their performance with that of equivalent voided columns. A nonlinear finite element parametric study was conducted using ABAQUS [22] to evaluate the effects of key design parameters on the structural response, including initial stiffness, load-carrying capacity, ductility, and residual strength. The parameters investigated include concrete compressive strength, longitudinal and transverse reinforcement ratios, void size, and cross-sectional shape.

The finite element model was validated against the results of an experimental investigation involving half-scale reinforced concrete columns with both solid and voided cross-sections. Details of the experimental program are presented in the following section. The primary novelty of this study lies in bridging the gap between experimental and numerical investigations by developing and validating finite element models capable of accurately simulating the behavior of voided reinforced concrete columns. In addition, the study presents a comprehensive parametric investigation that provides further insight into the structural behavior of these columns and the influence of the governing design parameters. The environmental benefits addressed in this study arise from the use of PET bottles solely as permanent void formers, offering a practical and sustainable approach for recycling plastic waste in reinforced concrete construction.

## 3. Experimental Program

The experimental program used to validate the numerical model consisted of testing 16 reinforced concrete columns, including 8 specimens with small cross-sections and 8 specimens with large cross-sections. Each cross-sectional category comprised four solid and four equivalent voided specimens. Within each category, each solid–voided pair was designed to investigate the effect of introducing voids while varying a specific design parameter. The first pair served as the control specimens, utilizing normal-strength concrete reinforced with four No. 12 longitudinal bars. The second pair incorporated an increased longitudinal reinforcement ratio by replacing the four No. 12 bars with four No. 16 bars. The third pair employed a reduced tie spacing of 50 mm compared with the 100 mm spacing used in the control specimens. The fourth pair utilized higher-strength concrete with a target compressive strength of 35 MPa instead of the 20 MPa used for the control specimens. The authors consider the number of specimens included in the experimental program to be sufficient for validating the finite element model, given the relatively large size of the tested columns and the broad range of parameters investigated. A detailed description of the experimental program and the corresponding test results is provided by Al Bayati and Tabsh [23].

**Figure 1:**
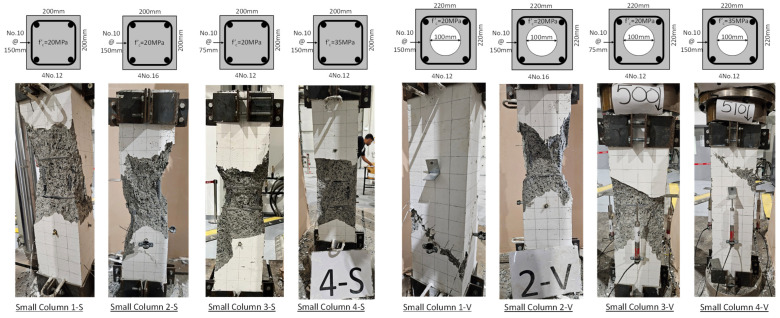
Details and photos of small column specimens used in the experimental study.

**Figure 2:**
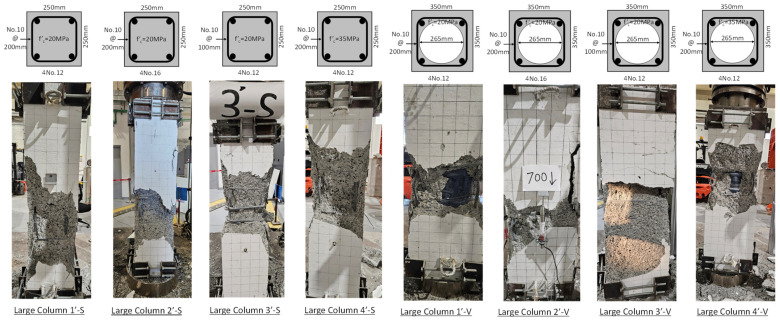
Details and photos of large column specimens used in the experimental study.

The four small solid columns were 900 mm long with square cross-sections measuring 200 mm × 200 mm, whereas the four small voided columns were 900 mm long with square cross-sections measuring 220 mm × 220 mm. Similarly, the four large solid columns were 1100 mm long with square cross-sections measuring 250 mm × 250 mm, while the four large voided columns were 1100 mm long with square cross-sections measuring 350 mm × 350 mm. In all specimens, a clear concrete cover of 25 mm was provided to the transverse reinforcement. Figure 1 and Figure 2 illustrate the geometry and reinforcement details of the tested columns and present representative photographs of the specimens after testing. In the voided specimens, the void occupied 16% of the total cross-sectional area of the small columns and 45% of that of the large columns. This percentage represents the reduction in concrete volume achieved through the incorporation of the voids. The cross-sectional dimensions of the specimens were selected such that each solid column and its corresponding voided counterpart contained the same amount of concrete and reinforcing steel. Consequently, both specimens had the same nominal axial compressive capacity in accordance with the provisions of the structural design code.

For the small voided columns, four commercially available PET bottles with a diameter of 100 mm were cut at their bases, nested inside one another, and manually compressed to form a stiff void capable of resisting deformation or cracking caused by the hydrostatic pressure of fresh concrete during casting. To ensure consistency, the diameter, length, and weight of the fabricated void were kept identical for all corresponding specimens. For the large voided columns, a single commercially available PET bottle with a diameter of 265 mm was used in each specimen. Prior to casting, strain gauges were attached to the longitudinal reinforcing bars to monitor strain development during testing. It should be noted that the sole purpose of the PET bottles was to serve as void formers, as their low strength and high flexibility prevented them from contributing to the axial load-carrying capacity of the columns. Once the void was established and the concrete had hardened, the PET bottles no longer played a structural role. Consequently, any long-term deterioration or disintegration of the PET bottles does not affect the structural behavior of the columns. Table 1 summarizes the details of the tested specimens, where *L* is the column length, *b* is the cross-sectional dimension of the square column, *f′_c_* is the concrete compressive strength, *s* is the tie spacing, *A_s_* is the area of the longitudinal reinforcement, and *D_v_* is the diameter of the void.

### 3.1. Material Properties

To determine the concrete compressive strength, twelve small-scale specimens were prepared from each concrete mix on the day of casting. Six specimens were 150 mm cubes, and the remaining six were 150 mm × 300 mm cylinders. The specimens were further divided into two groups corresponding to the target compressive strengths of 20 MPa and 35 MPa. All specimens were tested in compression using a universal testing machine.

A summary of the test results is presented in Table 2, which reports the measured compressive strengths for the concrete mixes with target strengths of 20 MPa and 35 MPa.

For the reinforcing steel, three 210 mm long specimens were prepared from each bar diameter (10 mm, 12 mm, and 16 mm), resulting in a total of nine test specimens. The specimens were subjected to uniaxial tensile testing using a Universal Testing Machine (UTM). The measured load and deformation data were subsequently used to develop the corresponding stress–strain curves and determine the mechanical properties of the reinforcing steel. Table 3 summarizes the steel properties obtained from the tensile tests. It should be noted that the reported strain-hardening strain corresponds to the onset of strain hardening of the material.

### 3.2. Instrumentation and Test Setup

Following concrete casting and curing, the column specimens were prepared for testing. The specimens were painted white, and a grid pattern was marked on their surfaces to facilitate crack observation and propagation monitoring. Small holes were then drilled into the columns to accommodate the screws and bolts used to secure the LVDTs and their supporting fixtures.

Prior to testing, steel bands were installed at both ends of each column to promote crack formation within the central region of the specimen, thereby ensuring accurate measurements from the LVDTs and strain gauges. Finally, each column was positioned in a 2500 kN-capacity Universal Testing Machine (UTM) in the Structural Engineering Laboratory at the American University of Sharjah, UAE, and subjected to monotonic axial compression under displacement-controlled loading at a rate of 0.3 mm/min.

## 4. Methodology

To provide a sustainable and efficient approach for investigating the behavior of voided reinforced concrete columns through parametric studies, and to further validate the analytical model and experimental findings, finite element models of the 16 tested columns were developed using the ABAQUS version 2024 software [22]. The finite element model enables future studies to investigate the influence of voids on columns with different dimensions, material properties, and void sizes beyond the scope of the experimental program.

The development of the finite element models involved five primary components: the concrete, longitudinal reinforcing bars, transverse reinforcement (stirrups), top steel loading plate, and end steel jackets. The concrete, steel plates, and steel jackets were modeled as three-dimensional deformable solid parts using extrusion geometry, whereas the longitudinal reinforcement and stirrups were modeled as three-dimensional deformable wire parts. Three material models were then defined to represent concrete, reinforcing steel, and elastic steel. After defining the material properties, the corresponding sections were created and assigned to the appropriate parts. Homogeneous solid sections were assigned to the concrete, steel plates, and steel jackets, while truss sections were assigned to the reinforcing bars and stirrups.

Three material definitions were considered in the analysis: nonlinear concrete for the column, nonlinear reinforcing steel for the longitudinal bars and stirrups, and linear elastic steel for the end plates and steel jackets. Since the structural response of the plates and jackets was not of interest in this study, they were modeled as linear elastic materials, whereas nonlinear constitutive models were adopted for both the concrete and reinforcing steel.

**Figure 3:**
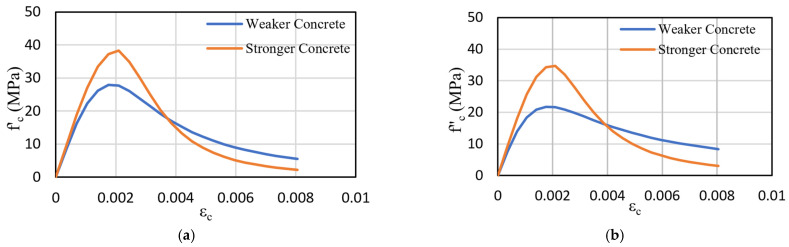
Concrete models used in the finite element analysis to validate the experimental results (**a**) small columns (**b**) large columns.

Two concrete grades were modeled in the finite element analysis: nominal 20 MPa concrete (actual compressive strengths of 28.15 MPa and 21.83 MPa) and nominal 35 MPa concrete (actual compressive strengths of 38.35 MPa and 35.02 MPa). The concrete density was taken as 2400 kg/m^3^. The elastic modulus was assumed to be 25,000 MPa for the nominal 20 MPa concrete and 27,000 MPa for the nominal 35 MPa concrete, while Poisson’s ratio was taken as 0.30 for both grades. The concrete plasticity parameters were adopted from values commonly reported in the literature for reinforced concrete column modeling [24,25]. The Thorenfeldt model [26] was employed to represent the nonlinear compressive behavior of both concrete grades because of its proven accuracy in predicting the stress–strain response, as illustrated in Figure 3.

The nonlinear concrete behavior in ABAQUS [22] was defined using the Concrete Damaged Plasticity (CDP) model. The required material parameters included the dilation angle, eccentricity, the ratio of biaxial to uniaxial compressive strength (Fbc/fb0), the shape factor (*K*), and the viscosity parameter. These parameters were taken as 3.6, 0.1, 1.16, 0.667, and 0.0001, respectively. The selected values were adopted from previous studies and were found to provide good agreement between the numerical predictions and the experimental results. Additional guidance on the finite element modeling of reinforced concrete members can be found in the recent work of Bolina and Rodrigues [27].

**Figure 4:**
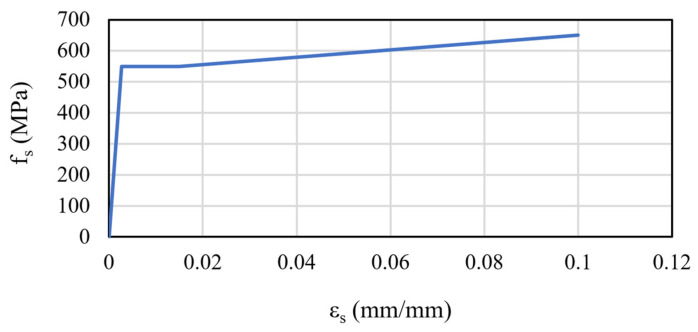
Steel model used in the finite element analysis to validate the experimental results.

The longitudinal reinforcing bars and transverse ties were assigned identical elastic and plastic material properties for all bar diameters, as the experimental tensile tests indicated that their mechanical properties were nearly identical. For the elastic behavior, a modulus of elasticity of 200 GPa and Poisson’s ratio of 0.30 were adopted. The nonlinear behavior of the reinforcing steel was represented using a simplified trilinear stress–strain model because of its simplicity and its ability to adequately capture the essential characteristics of the material response. A yield stress of 550 MPa was adopted based on the experimental results. The stress–strain relationship used to model the reinforcing steel is presented in Figure 4.

**Figure 5:**
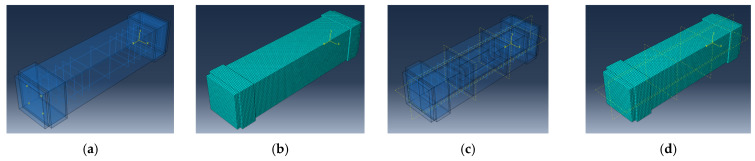
Finite element models (**a**) small columns model, (**b**) small columns mesh, (**c**) large columns model, and (**d**) large columns mesh.

After defining the material properties and assigning them to the corresponding sections, the Assembly module in ABAQUS was used to combine all model components into a single assembly. The individual parts were assembled to replicate the experimental setup as closely as possible, including the end steel plates and steel jackets. Typical finite element models of the solid and voided columns are presented in Figure 5. With respect to element selection and mesh generation, two element types were employed. The eight-node linear brick element with reduced integration (C3D8R) was used to model all three-dimensional solid components, including the concrete column, steel loading plates, and steel jackets. The longitudinal reinforcing bars and transverse ties were modeled using the two-node three-dimensional truss element (T3D2).

The PET bottles were not explicitly modeled in the finite element analysis. Instead, the voided columns were represented by introducing the corresponding void geometry without assigning material properties to the PET bottles, as their structural contribution was considered negligible. Owing to the relatively low stiffness of PET and the very small wall thickness of the bottles, their contribution to the stiffness and load-carrying capacity of the columns was assumed to be insignificant.

A uniform mesh size of 10 mm was adopted for all model components based on a mesh sensitivity analysis. Three mesh sizes (20 mm, 10 mm, and 5 mm) were evaluated. The 20 mm mesh produced results that differed from those obtained using the 10 mm mesh by approximately 20%, indicating insufficient accuracy. Although the 5 mm mesh improved the accuracy by only about 2% relative to the 10 mm mesh, it required approximately three times the computational time. Therefore, the 10 mm mesh was selected as an appropriate compromise between computational efficiency and solution accuracy. The adequacy of the selected mesh was verified by comparing the numerical responses obtained from the different mesh densities.

**Figure 6:**
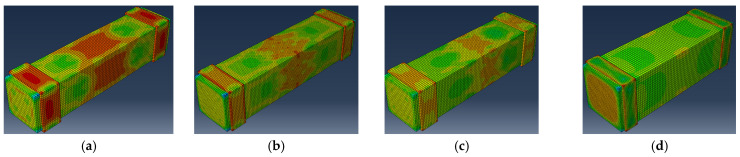
Axial stress contours. (**a**) small solid columns, (**b**) small void columns, (**c**) large solid columns, and (**d**) large void columns.

**Figure 7:**
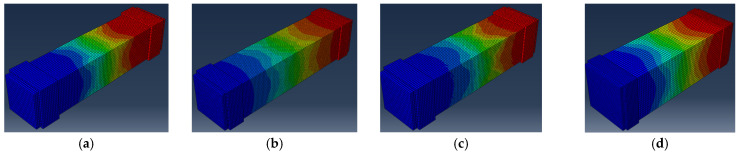
Axial displacement contours. (**a**) small solid columns, (**b**) small void columns, (**c**) large solid columns, and (**d**) large void columns.

Following the assembly of the model components, the interaction and loading conditions were defined. In the Interaction module, the longitudinal reinforcing bars and transverse ties were modeled using the Embedded Region Constraint, with the concrete designated as the host region and the reinforcement as the embedded region. The interfaces between the steel loading plates, steel jackets, and concrete were modeled using Tie constraints to replicate the experimental setup as closely as possible. In the Load module, an initial boundary condition was applied to the outer surface of the bottom steel plate to simulate a fixed support. A displacement-controlled loading condition was subsequently applied at the top steel plate to reproduce the experimental loading protocol. To accurately represent the symmetry conditions of the voided columns, roller boundary conditions were assigned to the planes of symmetry, thereby restricting displacement normal to the symmetry planes while allowing displacement in the remaining directions. A mesh size of 10 mm was adopted for the concrete, steel loading plates, and steel jackets. The longitudinal reinforcing bars and transverse ties were discretized using mesh sizes of 50 mm and 20 mm, respectively. Figure 6 presents the axial stress contours for the basic solid and voided columns with both small and large cross-sections. Figure 7 presents the corresponding axial displacement contours for the same specimens.

After validating the finite element model against the experimental results, a comprehensive parametric study was conducted to investigate the behavior of voided reinforced concrete columns with design parameters beyond the scope of the experimental program. The parameters considered include the cross-sectional shape, concrete compressive strength, longitudinal reinforcement diameter, tie spacing, and void diameter.

Table 4 summarizes all finite element models included in the parametric study, where h denotes the second dimension of the rectangular cross-section. The column identification (ID) follows the format FEM-X-Y-Z-W, where FEM denotes the finite element model, X represents the concrete compressive strength (*f′_c_*), Y represents the tie spacing (*s*), Z represents the longitudinal reinforcement bar diameter (*d_b_*), and W represents the void diameter within the column core (*D_v_*).

## 5. Results and Discussion

This section provides results of the finite element model validation with the experimental records and the subsequent outcome of the parametric study.

### 5.1. Validation of the Finite Element Model

**Figure 8:**
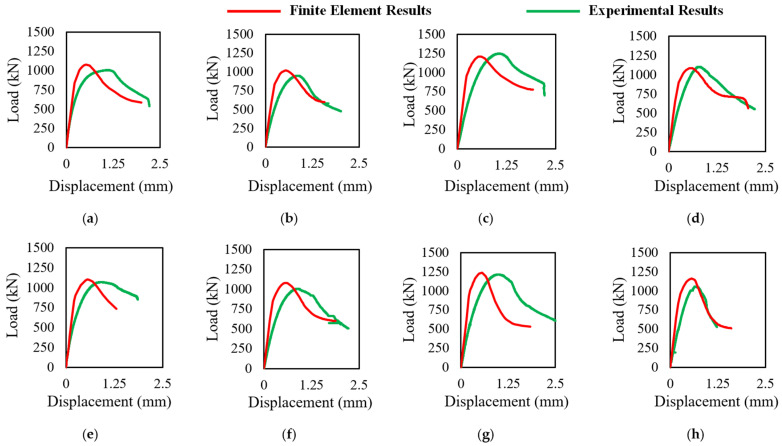
Comparison between finite element and experimental results for small columns. (**a**) 1-S, (**b**) 1-V, (**c**) 2-S, (**d**) 2-V, (**e**) 3-S, (**f**) 3-V, (**g**) 4-S, and (**h**) 4-V.

**Figure 9:**
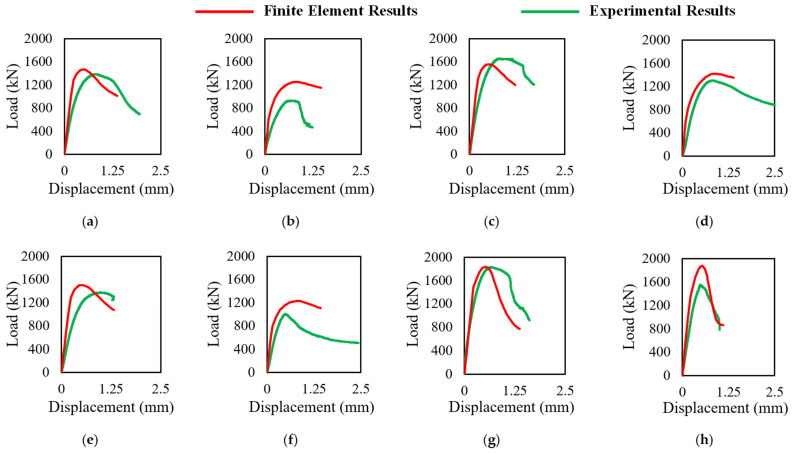
Comparison between finite element and experimental results for large columns. (**a**) 1′-S, (**b**) 1′-V, (**c**) 2′-S, (**d**) 2′-V, (**e**) 3′-S, (**f**) 3′-V, (**g**) 4′-S, and (**h**) 4′-V.

To ensure the reliability of the finite element model, it was directly compared with the experimental results. The ABAQUS software [22] was used to create models of the 16 columns, and load–displacement graphs were generated for each model to compare with the experimental data. Figure 8 and Figure 9 present the load–displacement graphs for all 16 column samples.

The load–displacement responses obtained from the finite element analysis generally showed good agreement with the experimental results. As expected, increasing the concrete compressive strength or the diameter of the longitudinal reinforcement resulted in a substantial increase in the load-carrying capacity, and these trends were accurately captured by the finite element models. Similarly, both the experimental and numerical results indicated that the influence of tie spacing on the structural response was relatively small.

Overall, the predicted ultimate axial compressive capacities agreed well with the experimental values, with differences of less than 10% for all specimens except 1′-V, 3′-V, and 4′-V, for which the discrepancies were 28%, 22%, and 20%, respectively. The larger deviations may be attributed to the idealized assumptions adopted in the finite element model [28] and higher uncertainties associated with experimental testing. In particular, the concrete was modeled as a homogeneous material with uniform properties throughout the column, whereas actual concrete inevitably exhibits material heterogeneity and localized imperfections. The influence of these effects becomes more pronounced in the large voided columns, where the void occupies a significant portion of the cross-section. In such specimens, the remaining concrete surrounding the void is relatively thin and less confined, making the structural response more sensitive to local variations in concrete properties. Consequently, small material inconsistencies that are not represented in the finite element model may lead to noticeable reductions in the experimentally measured load-carrying capacity. It is also noteworthy that the three specimens exhibiting the largest discrepancies between the experimental and numerical results all contained large voids occupying most of the concrete core, causing a greater proportion of the axial load to be resisted by the concrete cover and the longitudinal reinforcement.

Another observation from the load–displacement curves is that the finite element models generally exhibited higher initial stiffness than the experimental specimens, indicating a slightly stiffer structural response. This overestimation of the initial stiffness is primarily attributed to the idealized assumptions adopted in the finite element modeling, including the omission of initial geometric imperfections, the simplified representation of concrete tension stiffening, the assumption of perfect bond between the reinforcing steel and concrete, and idealized boundary conditions. These assumptions result in a stiffer numerical response than that observed experimentally from laboratory testing, where material heterogeneity, initial imperfections, bond-slip effects, and support compliance contribute to increased deformation and reduced stiffness.

### 5.2. Parametric Study

The results of the parametric study are presented through load–displacement relationships to evaluate the sensitivity of the structural response to variations in the investigated design parameters. This approach allows for a clearer assessment of the influence of each variable on the overall performance of the columns.

For the parametric study, the strength of a specimen is defined as the peak load attained from the load–displacement curve. The ductility index is defined as the ratio between the displacement corresponding to 85% of the peak load in the post-peak region and the corresponding displacement at 85% of the peak load in the pre-peak region. The initial stiffness is calculated as the slope of the load–displacement curve between the origin and the point corresponding to 50% of the peak load. The residual strength is defined as the ratio of the residual load at a displacement equal to twice the displacement at peak load to the maximum load attained.

#### 5.2.1. Effect of Concrete Compressive Strength

**Figure 10:**
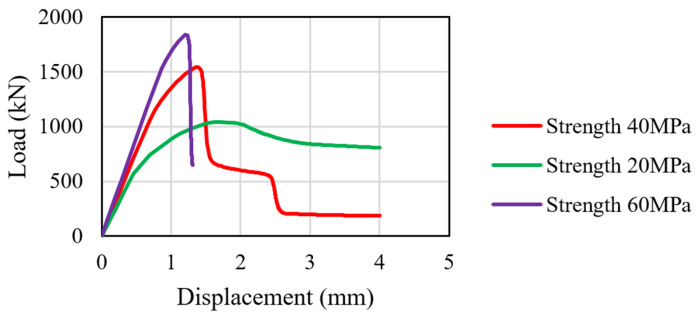
Finite element parametric study on effect of changing concrete compressive strength.

**Figure 11:**
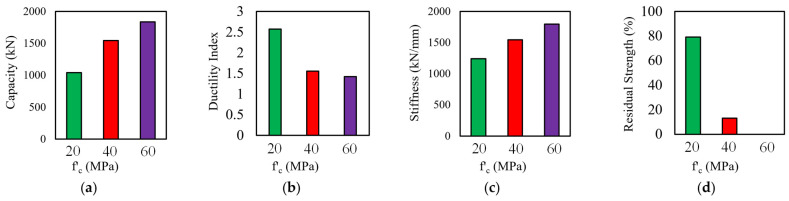
Effect of changing concrete compressive strength on (**a**) load carrying capacity, (**b**) ductility, (**c**) stiffness, and (**d**) residual strength.

The effect of concrete compressive strength on the behavior of voided reinforced concrete columns was investigated by considering three concrete strength levels (f′_c_): 20 MPa, 40 MPa, and 60 MPa. Accordingly, three finite element models were developed, with each model incorporating a different concrete compressive strength while keeping all other parameters constant. Figure 10 and Figure 11 present the corresponding load–displacement responses and a comparison of the ultimate axial load capacities, respectively.

As expected, increasing the concrete compressive strength resulted in an increase in the axial load-carrying capacity of the voided columns. This behavior is expected because the concrete compressive strength directly contributes to the axial resistance of reinforced concrete columns and represents a fundamental parameter in structural design equations. Increasing the concrete compressive strength from 20 MPa to 40 MPa increased the ultimate load capacity from 1041 kN to 1545 kN, corresponding to a 48.4% increase. However, increasing the compressive strength from 40 MPa to 60 MPa resulted in a smaller increase in capacity, from 1545 kN to 1836 kN, representing a 19% improvement. The reduced rate of increase at higher strength levels is consistent with the diminishing contribution of concrete strength to the overall axial capacity, resulting in a tendency toward reduced efficiency gains as the concrete strength increases.

The ductility indices of the finite element models were also evaluated and are presented in Figure 11. The calculated ductility indices for the 20 MPa, 40 MPa, and 60 MPa concrete models were 2.50, 1.55, and 1.47, respectively. These results indicate a reduction in ductility with increasing concrete compressive strength. This behavior agrees with the well-established observation that high-strength concrete exhibits a more brittle response and reduced post-peak deformation capacity compared with normal-strength concrete.

The influence of concrete compressive strength on the initial stiffness was also investigated. The results showed that the initial stiffness increased with increasing concrete strength, reaching values of 1243 kN/mm, 1545 kN/mm, and 1796 kN/mm for the 20 MPa, 40 MPa, and 60 MPa models, respectively. This increase is attributed to the higher modulus of elasticity associated with higher-strength concrete, which enhances the resistance of the column to deformation under applied compressive loads.

Finally, the effect of concrete compressive strength on the residual strength was investigated. The results indicated a significant reduction in residual strength as the concrete strength increased. The residual strength ratios for the 20 MPa, 40 MPa, and 60 MPa models were 80%, 13%, and 0%, respectively. The 20 MPa model maintained a substantial portion of its load-carrying capacity after reaching the peak load, demonstrating a more gradual post-peak degradation. In contrast, the 40 MPa model exhibited a significant reduction in strength beyond the peak load, while the 60 MPa model experienced an abrupt loss of load-carrying capacity immediately after reaching its ultimate capacity, resulting in negligible residual strength. This behavior can be attributed to the increased brittleness associated with higher-strength concrete, which leads to more rapid crack propagation and crushing after peak load. Consequently, the ability of the column to sustain additional deformation and carry load in the post-peak region is significantly reduced.

#### 5.2.2. Effect of Longitudinal Rebars Size

**Figure 12:**
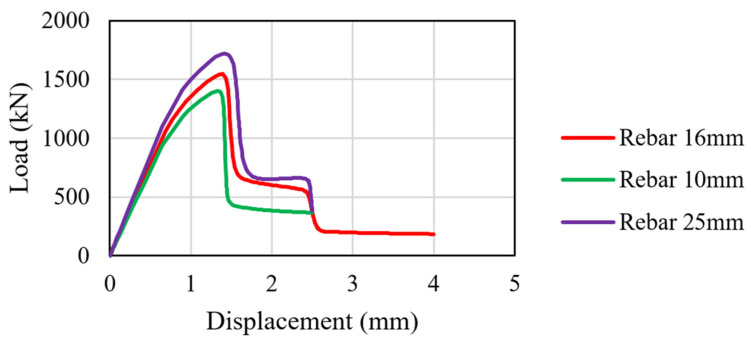
Finite element parametric study on effect of changing longitudinal reinforcement ratio.

**Figure 13:**
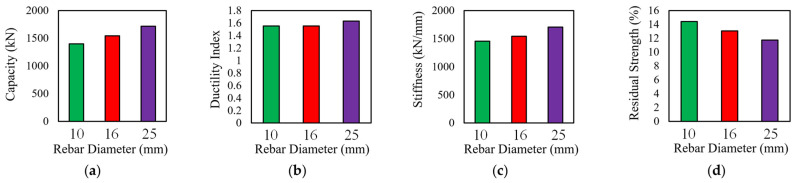
Effect of changing longitudinal reinforcement ratio on (**a**) load carrying capacity, (**b**) ductility, (**c**) stiffness, and (**d**) residual strength.

The effect of longitudinal reinforcement bar diameter was investigated as the next parameter. Three finite element models were developed to evaluate this effect: the reference model containing 16 mm diameter bars and two additional models incorporating 10 mm and 25 mm diameter bars. Figure 12 and Figure 13 present the corresponding load–displacement responses and ultimate load capacities, respectively.

The results indicate that increasing the diameter of the longitudinal reinforcement enhances the ultimate axial load-carrying capacity of the column. The ultimate loads obtained for the models with 10 mm, 16 mm, and 25 mm reinforcement bars were 1400 kN, 1550 kN, and 1720 kN, respectively. Increasing the bar diameter from 10 mm to 16 mm and from 16 mm to 25 mm resulted in increases in ultimate capacity of approximately 10% and 11%, respectively. This behavior agrees with fundamental structural mechanics principles and is consistent with the contribution of longitudinal reinforcement considered in the axial capacity equation of ACI 318 [29].

The ductility index was only slightly influenced by changes in reinforcement diameter. The models containing 10 mm and 16 mm bars exhibited identical ductility indices of 1.55, whereas increasing the bar diameter to 25 mm resulted in a marginal increase to 1.63. These results indicate that increasing the reinforcement ratio provides only a limited improvement in the post-peak deformation capacity of the voided columns.

The influence of reinforcement diameter on initial stiffness was also evaluated. The calculated stiffness values for the models with 10 mm, 16 mm, and 25 mm bars were 1456 kN/mm, 1545 kN/mm, and 1707 kN/mm, respectively. The increase in stiffness is attributed to the greater contribution of the longitudinal reinforcement to resisting axial deformation, resulting in a steeper load–displacement response.

Finally, the residual strength ratios of the three models are presented in the corresponding figure. The results show a slight reduction in residual strength ratio with increasing reinforcement diameter, with values of 14.4%, 13.0%, and 11.75% for the models with 10 mm, 16 mm, and 25 mm bars, respectively. This trend is primarily attributed to the substantial increase in ultimate load capacity, while the residual load values remained relatively comparable among the three models. Since the residual strength ratio is defined as the residual load divided by the ultimate load, an increase in ultimate capacity results in a reduction in this ratio even when the residual load remains nearly unchanged.

#### 5.2.3. Effect of Tie Spacing

**Figure 14:**
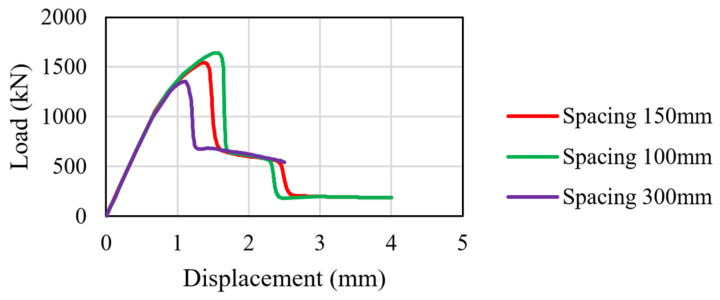
Finite element parametric study on effect of changing transverse reinforcement ratio.

**Figure 15:**
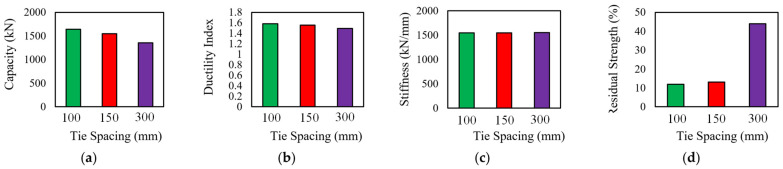
Effect of changing transverse reinforcement ratio on (**a**) load carrying capacity, (**b**) ductility, (**c**) stiffness, and (**d**) residual strength.

The effect of tie spacing was investigated as the next parameter. Three finite element models were developed to evaluate its influence on the behavior of voided reinforced concrete columns, with tie spacings of 100 mm, 150 mm, and 300 mm. The model with a tie spacing of 150 mm was selected as the reference model. Figure 14 and Figure 15 present the load–displacement responses and a comparison of the ultimate load capacities of the three models, respectively.

The results indicate that increasing the tie spacing reduces the axial load-carrying capacity of the columns. The ultimate loads obtained for tie spacings of 100 mm, 150 mm, and 300 mm were 1640 kN, 1550 kN, and 1355 kN, respectively. Increasing the tie spacing from 100 mm to 150 mm resulted in a 6% reduction in capacity, while a further increase from 150 mm to 300 mm produced an additional 12% reduction. This behavior is expected because larger tie spacing decreases the amount of transverse reinforcement and reduces the confinement provided to the concrete core. Although confinement is not directly included in the axial capacity equation, reduced confinement leads to increased lateral expansion of the concrete, greater vulnerability of the longitudinal reinforcement to buckling, and earlier development of transverse cracking, all of which contribute to a reduction in the ultimate load capacity.

A similar trend was observed for ductility. The ductility indices for the models with tie spacings of 100 mm, 150 mm, and 300 mm were 1.58, 1.55, and 1.49, respectively. The reduction in ductility with increasing tie spacing can be attributed to the reduced confinement level and the increased unsupported length of the longitudinal reinforcement between adjacent ties. Consequently, the columns exhibit a more brittle response and reduced post-peak deformation capacity.

The influence of tie spacing on the initial stiffness was also evaluated. The results showed that tie spacing had a negligible effect on the initial stiffness, with all three models exhibiting approximately the same stiffness value of 1545 kN/mm. This behavior is expected because the initial stiffness is primarily governed by the elastic properties of the concrete and longitudinal reinforcement and is less dependent on the amount of transverse reinforcement.

Finally, the effect of tie spacing on residual strength was investigated. The residual strength ratios for the models with tie spacings of 100 mm, 150 mm, and 300 mm were 11.9%, 13.1%, and 44%, respectively. The small difference between the 100 mm and 150 mm models is attributed to their similar post-peak responses. The relatively high residual strength ratio obtained for the 300 mm model is a consequence of the adopted residual strength definition, which considers the residual load at a displacement equal to twice the displacement at peak load. Because this parameter depends on both the selected displacement criterion and the shape of the post-peak load–displacement response, alternative definitions (e.g., using 1.5 times the displacement at peak load instead of 2.0 times) may lead to different trends. From an overall practical perspective, Figure 14 indicates that the three models exhibit comparable residual behavior when displacements beyond approximately 2.3 mm are excluded.

#### 5.2.4. Effect of Void Diameter

**Figure 16:**
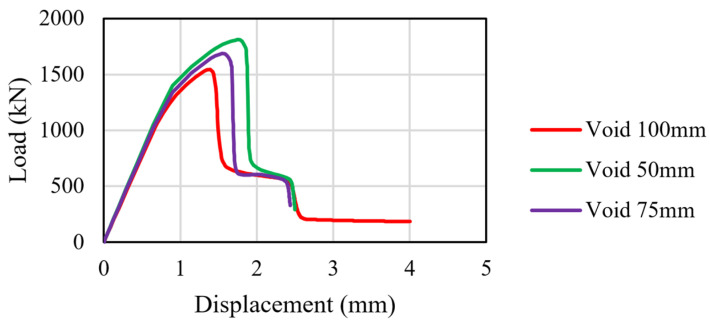
Finite element parametric study on effect of changing void diameter.

**Figure 17:**
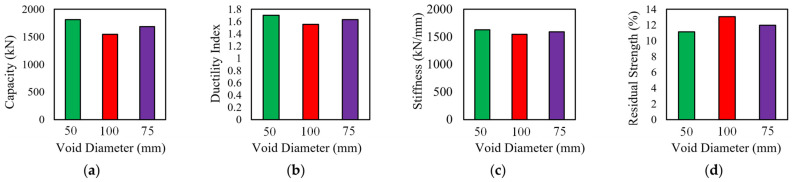
Effect of changing void diameter on (**a**) load carrying capacity, (**b**) ductility, (**c**) stiffness, and (**d**) residual strength.

The effect of void diameter was investigated as the next parameter. Three finite element models were developed: the reference model with a void diameter of 100 mm and two additional models with void diameters of 75 mm and 50 mm. Figure 16 and Figure 17 present the corresponding load–displacement responses and ultimate load capacities, respectively.

The results indicate that increasing the void diameter reduces the axial load-carrying capacity of the columns. The ultimate load capacities for the models with void diameters of 50 mm, 75 mm, and 100 mm were 1811 kN, 1687 kN, and 1545 kN, respectively. Increasing the void diameter from 50 mm to 75 mm resulted in a 7.5% reduction in capacity, while a further increase from 75 mm to 100 mm produced an additional 9.2% reduction. This behavior is expected because larger voids reduce the effective concrete area and decrease the size of the confined concrete core. As a result, less concrete is available to resist the applied axial load, leading to a reduction in the overall load-carrying capacity.

A similar trend was observed for ductility. The ductility indices obtained for the 50 mm, 75 mm, and 100 mm void diameter models were 1.70, 1.63, and 1.55, respectively. Although the reduction was relatively limited, the results indicate that increasing the void diameter slightly decreases the ductility of the columns. This behavior can be attributed to the reduction in the confined concrete region, which increases the likelihood of brittle behavior and reduces the post-peak deformation capacity.

The influence of void diameter on initial stiffness was also evaluated. The results showed a slight reduction in stiffness with increasing void diameter, with values of 1630 kN/mm, 1591 kN/mm, and 1544 kN/mm for the 50 mm, 75 mm, and 100 mm void diameter models, respectively. This decrease is attributed to the reduction in effective concrete area and the corresponding reduction in the stiffness contribution of the concrete. However, the relatively small differences indicate that void diameter has a limited influence on the initial stiffness within the investigated range.

Finally, the effect of void diameter on residual strength was examined. The residual strength ratios for the models with void diameters of 50 mm, 75 mm, and 100 mm were 11%, 12%, and 13%, respectively. The slight increase in residual strength ratio with increasing void diameter is primarily related to the normalization procedure used to calculate this parameter. Since the residual load levels in the post-peak region were relatively similar among the models, the models with higher ultimate capacities resulted in lower residual strength ratios when the residual load was divided by the larger peak load. Therefore, the observed increase in residual strength ratio does not indicate improved post-peak behavior, but rather reflects the influence of the selected residual strength definition.

#### 5.2.5. Effect of Cross-Section Shape

**Figure 18:**
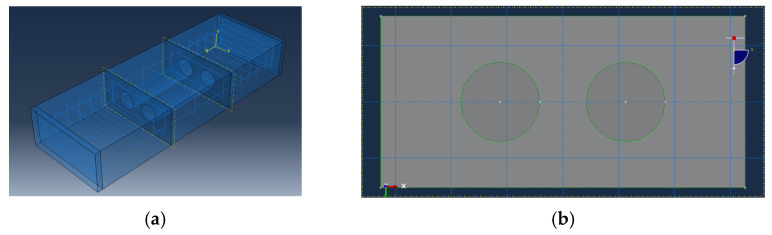
(**a**) ABAQUS rectangular section model; (**b**) Voids within the rectangular model.

The final parameter investigated was the influence of cross-sectional shape. Two finite element models were developed for this purpose. The first model was the reference case, consisting of a square cross-section measuring 220 × 220 mm^2^ with a centrally located circular void of 100 mm diameter. The second model represented a rectangular cross-section measuring 320 × 150 mm^2^ and containing two circular voids, each with a diameter of 70 mm. The dimensions of the rectangular section were selected such that its gross cross-sectional area was identical to that of the square section, ensuring a consistent basis for comparison between the two models. Figure 18 presents the cross-sectional geometry and the corresponding ABAQUS finite element model of the rectangular column.

**Figure 19:**
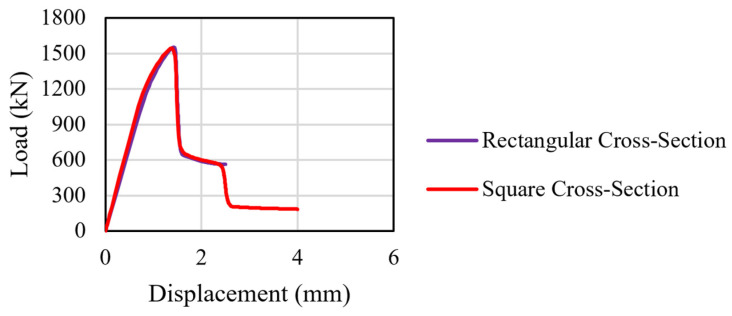
Effect of changing cross-section shape on load–displacement relationship.

Figure 19 presents the load–displacement responses of the square and rectangular models. The results indicate that both models exhibited nearly identical behavior, with comparable initial stiffness, ultimate load capacity, and post-peak response. This similarity is expected because the two models were designed to have the same concrete area and longitudinal reinforcement ratio. More specifically, the load-carrying capacity, ductility index, initial stiffness, and residual strength were identical for both models, with corresponding values of 1550 kN, 1.55, 1545 kN/mm, and 13%, respectively. These results suggest that, within the limited configurations investigated in this study, changing the cross-sectional shape from square to rectangular while maintaining the same concrete area and reinforcement quantity has a negligible influence on the axial compression behavior of the voided columns. However, further investigation involving additional cross-sectional geometries, such as circular sections and rectangular sections with different aspect ratios, is required before drawing a general conclusion regarding the influence of cross-sectional shape.

## 6. Conclusions

This study employed a validated finite element model to investigate the feasibility of using Polyethylene Terephthalate (PET) bottles as void formers in non-slender reinforced concrete columns subjected to concentric axial compression. The proposed approach reduces concrete consumption in columns without significantly compromising the structural performance. The finite element model was validated using experimental results from 8 solid and 8 voided reinforced concrete columns with varying longitudinal reinforcement ratio, tie spacing, void size, and concrete compressive strength.

The following conclusions can be drawn from this study:

1. Embedding stacked PET bottle voids within the core of reinforced concrete tied columns did not adversely affect the structural response under axial compression, as the behavior of voided columns remained comparable to that of solid columns.
2. The developed nonlinear finite element model showed excellent agreement with experimental results, albeit showing consistently higher stiffness than its equivalent experimental outcome. It proved to be a reliable tool for predicting the behavior of both solid and voided columns under concentric compressive loads.
3. The finite element-based parametric study provided valuable insight into the influence of column geometry, reinforcement ratios, concrete strength, and void configuration on the structural performance of voided columns, including load capacity, stiffness, and ductility.
4. Increasing the concrete compressive strength from 20 MPa to 40 MPa and 60 MPa increased the load-carrying capacity by 48% and 19%, respectively, and increased the initial stiffness by 24% and 16%, respectively, while reducing ductility by 38% and 5%.
5. Increasing the longitudinal reinforcement bar diameter from 10 mm to 16 mm and 25 mm enhanced the load capacity by 10% and 11%, respectively, and increased stiffness by 6% and 10%, respectively, with only minor changes in ductility (0% and 5%).
6. Increasing tie spacing from 100 mm to 150 mm and 300 mm reduced the load-carrying capacity by 6% and 12%, respectively, with no influence on stiffness and limited effect on ductility (2% and 4%).
7. Increasing the void diameter from 50 mm to 75 mm and 100 mm resulted in moderate reductions in column strength (7% and 9%), insignificant impact on stiffness (2% and 3%), and small change in ductility (4% and 5%).
8. Changing the column shape while maintaining the same cross-sectional area had no influence on load capacity, stiffness, ductility, and residual strength.

## Figures and Tables

**Table 1:** Details of the specimens considered in the experimental program [23].

Column Label	*L* (mm)	*b* (mm)	Actual *f′_c_* (MPa)	*s* (mm)	*A_s_* (mm^2^)	*D_v_* (mm)
1-S	900	200	28.15	150	452	-
1-V	900	220	28.15	150	452	100
2-S	900	200	28.15	150	804	-
2-V	900	220	28.15	150	804	100
3-S	900	200	28.15	75	452	-
3-V	900	220	28.15	75	452	100
4-S	900	200	38.35	150	452	-
4-V	900	220	38.35	150	452	100
1′-S	1100	250	21.83	200	452	-
1′-V	1100	350	21.83	200	452	265
2′-S	1100	250	21.83	200	804	-
2′-V	1100	350	21.83	200	804	265
3′-S	1100	250	21.83	100	452	-
3′-V	1100	350	21.83	100	452	265
4′-S	1100	250	35.02	200	452	-
4′-V	1100	350	35.02	200	452	265

**Table 2:** Actual compressive strength of the concrete used in the experimental program [23].

Column Type	Average Compressive Strength of 20 MPa Concrete (MPa)		Average Compressive Strength of 35 MPa Concrete (MPa)	
	Cube	Cylinder	Cube	Cylinder
Small Columns	32.36	28.15	42.04	38.35
Large Columns	24.62	21.83	39.06	35.02

**Table 3:** Actual properties of the steel used in the experimental program [23].

Rebar Size (mm)	Average Yield Stress (MPa)	Average Ultimate Stress (MPa)	Average Modulus of Elasticity (GPa)	Average Strain Hardening (mm/mm)
No 10	610.73	726.81	197.20	0.0152
No. 12	551.53	647.30	216.34	0.0157
No. 16	559.31	647.27	195.05	0.0154

**Table 4:** Characteristics of the columns considered in the finite element-based parametric study.

Column ID	*L* (mm)	*b* (mm)	*h* (mm)	*f′_c_* (MPa)	*s* (mm)	*A_s_* (mm^2^)	*D_v_* (mm)
FEM-40-150-16-100	900	220	220	40	150	804	100
FEM-20-150-16-100	900	220	220	20	150	804	100
FEM-60-150-16-100	900	220	220	60	150	804	100
FEM-40-150-10-100	900	220	220	40	150	314	100
FEM-40-150-25-100	900	220	220	40	150	1963	100
FEM-40-100-16-100	900	220	220	40	100	804	100
FEM-40-300-16-100	900	220	220	40	300	804	100
FEM-40-150-16-100	900	220	220	40	150	804	50
FEM-40-150-16-100	900	220	220	40	150	804	75
FEM-Rectangular Section	900	320	150	40	150	804	67.5

## Data Availability

The original contributions presented in this study are included in the article. Further inquiries can be directed to the corresponding author.
